# Universal Health Coverage Policy and Progress towards the Attainment of Universal Sexual and Reproductive Health and Rights Services in Ethiopia

**DOI:** 10.4314/ejhs.v32i1.19

**Published:** 2022-01

**Authors:** Yifru Berhan, Mahbub Ali, Awoke Tassew, Akiyo Nonogaki

**Affiliations:** 1 St Paul's Hospital Millennium Medical College; 2 UNFPA, Ethiopia

## Abstract

Critical interpretive analysis of literature review was applied to shed light on the status of universal access to Sexual and Reproductive Health and Rights (SRHR) and the progress towards Universal Health Coverage (UHC) in Ethiopia. Special emphasis was given to the determinations of the Ethiopian health policy frameworks to include comprehensive SRHR services in the UHC benefit package. Clinical services for pregnant women and newborn, abortion care, family planning, Female Genital Mutilation (FGM) complication treatment, Comprehensive Sexuality Education (CSE), and sexual health services are included in the national cost exempted services, but the latter three are not yet included in the health programs with defined objective and work plan. Capital intensive Sexual and Reproductive Health (SRH) services (such as infertility and reproductive cancers diagnosis and treatment) are not included in the UHC benefit package. Over the last two decades, a substantive progress is made in family planning service and maternal and child health, probably because they were taken as Millennium Development Goals (MDGs) indicators and have got better financial protection and political commitment. In order to include other SRHR services in the benefit package in due course and attain universal SRHR services without financial hardship in the Primary Health Care (PHC) setting, the domestic financing should be endorsed as a driving force. To make the multi-sectoral efforts towards achieving UHC and sustainable development goals (SDGs) complete, building resilient health systems through the humanitarian-development nexus for health systems strengthening in fragile setting should be equally prioritized, thereby leaving no one behind underserved.

## Introduction

At present, universal health coverage (UHC) is a global approach to improving the health service utilization of the people and ensuring equity. The primary aim of UHC is providing essential health service to all people who need it, without suffering from financial hardship (1). As UHC is one of the pillars of sustainable development goals (SDGs), universal access to Sexual and Reproductive Health and Rights (SRHR) is also an integral part and major component of UHC (2).

The concept of UHC was in the Alma-Ata declaration (1978) and Global Health Strategy for Health for All by the year 2000 (1979) (3). However, the UHC approach has got much popularity after it was included in the World Health Report by the World Health Organization (WHO) in 2010 (1). Then after, achieving UHC is not only accepted as the most preferred approach for ensuring equitable and accessible health services, but also one of the global priorities for multidimensional development.

The health aspect of the SDGs aspires to make UHC with financial protection in place for all who need the service (2). This is primarily because of the determination of attaining ‘health for all’ by 2030. According to the WHO, “investments in UHC and the other SDG targets could prevent 97 million premature deaths globally by 2030 and add as much as 8.4 years of life” (4). To capitalize the transformative ideas within the clauses of the UHC, the United Nations (UN) passed a political declaration on UHC in 2019, primarily reaffirming their commitment to achieve UHC by 2030 (5). This declaration underpinned UHC as an important undertaking for the health and wellbeing of the people, and as the best strategy (among others) to achieve gender equality and women's empowerment (5).

Although UHC is a popular and value-based strategy to make high-quality, integrated, and people-centered health services that is accessible to everyone without incurring financial hardship, the gap in the coverage between low-and high-income (that target) is still wide. The global UHC effective coverage index in 2019 stands at 60.3 on a scale of 0–100, while the mean was 43.9 in sub-Saharan Africa and 47 in Ethiopia (6). A study showed that the overall UHC of Ethiopia was about 34% in 2015 (7), which is expected to be 80% by 2030. Despite the UHC has increased by approximately 20% between 2000 and 2015 globally, half of the global population still lacks the coverage of essential health services (8). The wide-gap between high-and low-income countries is not only because of economic disparity, but also due to weak health system and humanitarian crisis, unless otherwise the latter two are partly determined by the economic status. The purpose of this review was to identify the progress, gaps, and key Ethiopia-specific actions to make SRHR part of the UHC benefit package by applying a critical interpretive analysis method.

## Literature Search Strategy

In this review, ‘critical interpretive synthesis was applied. Data search strategy was computer based for literature on current and proposed UHC and SRHR policy documents, roadmaps, and peer reviewed articles/scientific papers. For policy and roadmap documents, normative evidence from the Ethiopian Ministry of Health (MOH), UNFPA, UNICEF, WHO, World bank, Global Fund, UNAIDS, and other credible sources were searched. Both peer reviewed research articles and gray literature and policy documents were included. Peer reviewed articles were further searched from MEDLINE/PUBMED, Cochrane library, HINARI, EMBASE, Google, Google scholar, and AJOL databases. The literature search was further strengthened by searching the relevant literature from the reference lists of retrieved articles.

Search terms used were: universal health coverage, UHC, UHC health benefit package, sexual and reproductive health and rights, SRHR, SRH, comprehensive SRHR, universal SRHR, sexual health, sexual rights, reproductive health, reproductive rights, policy, road map, financial protection, and health expense. Emphasis was given to national policy and strategic plan documents, UN and UN agencies policy documents, local and regional research data.

## Global and Regional Efforts in Embedding SRHR Services in the UHC Benefit Package

In recent years, the UHC has got the attention of the UN, national policy makers and decision makers globally, largely because of the inclusive principle it is derived from and stands for (9). This leads to the understanding that getting essential health service, when needed, is part and parcel of the fundamental principle of human rights (‘the right to health’) that cannot be restricted or denied because of financial constraint, inequality, and minorities within the social stratum, which is also stated in the Universal Declaration of Human Rights. This guiding principle has embedded SRHR in the UHC benefit package.

The decisions made by 179 governments on women's SRHR during the International Conference on Population and Development (ICPD) in Cairo in 1994 and ICPD25 in Nairobi in 2019 are considered as milestones to revolutionize reproductive health policies and programs of action, by revitalizing the governments' commitment to the accomplishment of universal access to SRHR services through UHC (10).

There is a common understanding that the progress and the success of UHC (thereby the SDG) cannot be thought and attained without parallel achievement of universal SRHR. The SDG 3 on good health and wellbeing and SDG 5 on gender equality can be achieved when the SRHR is integrated to UHC national and regional plans and implementations, as SRHR and UHC are mutually reinforcing the progress towards the SDGs.

However, there is a paucity of evidence that has assessed the level of integration and the challenges encountered to include comprehensive SRH services within the national policies, plans, and processes for UHC. From anecdotal evidence, the progress towards attaining the universal SRHR by 2030 was not universally on track. The East African countries, in particular, are in the lower range of achievement (11–13). The exemplary performance and good progress in achieving universal SRHR services by some of the low and middle-income countries (like Thailand, Uruguay, Cambodia) (14) is an impetus for East African countries to take it as an important undertaking. In general, the current momentum around UHC provides an opportunity to progressively include and integrate comprehensive SRHR within the country-specific ‘UHC benefit package’, ‘Pool Health Financing’, and ‘Financial protection’.

## Components of Comprehensive SRHR Specific UHC Benefit Package (15)

1. Providing skilled service to pregnant, delivering and postpartum women, including care for newborn and women with fistula;
2. Counseling and providing modern contraception methods to prevent unplanned pregnancy and increase birth spacing;
3. Providing safe abortion and treating unsafe abortions and potential complications;
4. Preventing and treating HIV, Sexually Transmitted Infection (STI) and Reproductive Tract Infection (RTI);
5. Preventing, screening/early detecting, and treating reproductive cancers (especially cervical and breast cancers);
6. Counseling and providing sub-fertility and infertility treatment;
7. Preventing, detecting, and treating sexual and Gender-Based Violence (GBV) and other harmful traditional practices such as Female Genital Mutilation (FGM) and child marriage;
8. Providing Comprehensive Sexuality Education (CSE); and,
9. Counseling and providing services for Sexual Health and Wellbeing, including Menstrual Health Management (MHM).

Providing CSE is to prevent sexual violence, child marriage, STI (including HIV), increase contraceptive utilization to prevent unplanned pregnancy, unsafe abortion, and reduce the total fertility rate (16). Similarly, counseling and providing services for sexual health and wellbeing give an opportunity to detect and integrate sexual disharmony with FGM, STI, and GBV; to counsel on contraceptive use, fertility treatment, antenatal care, and screening for reproductive cancers.

In addition to the immediate impact of the integrated SRHR preventive, rehabilitative, and therapeutic services, some of the interventions may have a lifelong impact. For instance, preventing FGM is preventing lifelong physical scar, sexual and mental health problems and disorders. Likewise, unprotected sexual intercourse during the age of adolescence is known to increase the risk of unplanned pregnancy, unsafe abortion, and HIV/STI as immediate consequences, and infertility and developing cervical cancers in later life (17).

Some of the SRH services are linked to human rights issues. Providing contraceptive methods for a woman of her choice or providing safe abortion as she requests is respecting her rights and ensuring women's empowerment. Furthermore, building the decision-making power of women on her body and her daughter's body by providing CSE and changing the patriarchal norm is one step forward to eliminate FGM, child marriage, gender inequality, and other gender-based discrimination and rights violations.

In Ethiopia, a large body of literature (7,10,11), including Ethiopian Demographic and Health Surveys (EDHS) (18) and the UNFPA Strategic Plan (2018–2021) (19), has noted that good progress of some of SRHR indicators (maternal health, family planning, and HIV treatment) has been made over the decades. However, gender inequalities and violating the girls and women's rights are still incomparably prevalent, as quantified by the high prevalence of FGM, child marriage, marriage by abduction, polygamy and polygyny, wife beating, gender stereotyping, lip disks, bush delivery, and many more (20).

**Figure 1:**
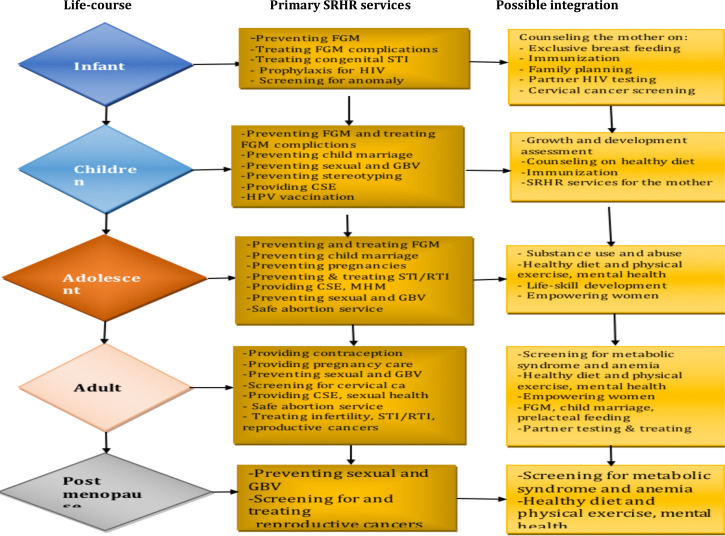
The age-specific SRHR services and potential integration. The vertical arrows are put just to show the need for the continuity of the SRHR services along the life-course. POP = Pelvic Organ Prolapse

Universal access to SRHR services can be achieved when all components are addressed and integrated with the health system at all portals of access in all areas and segments of the population. Understanding the interlinkages between the different components of the SRHR package at the different life-course is essential for planning and implementing services integration, and for each to have a multiplied effect (Figure 1).

The concept of integration in this scenario is not only providing or linking to non-SRHR health services, but also integrating with other SRHR services within the box. Therefore, the primary integration of services focuses on the SRHR domain, and secondary integration or linkage can be still into SRHR related or unrelated but important health services (Annex 1).

## Overview of the Ethiopia Health Policy Supporting the Progress Towards UHC

Little is known about the Ethiopian health policy towards UHC in the 1980s; as a WHO member country, however, the Alma-Ata declaration was endorsed. Basing on the 1993 health policy, Ministry of Health (MOH) had launched the 20 years National Health Sector Development Program (HSDP I-IV) and concluded in 2015 (21), at which time the health sector transformation plan (HSTP I) was born and soon commenced as national health strategic plan (22). During the era of the HSDP, the concept of UHC was expressed in different forms (availing essential drugs and supplies, accessing to health services and health workers, and the marginal budgeting for bottlenecks to reach the MDGs).

UHC was envisaged by HSDP IV and capitalized by the HSTP I to be a goal for Ethiopia's health sector in the decades to come. It was also the mid-term review of the HSDP IV that let the MOH be embarked on developing another 20-year Health Sector Transformation Roadmap titled, “Envisioning Ethiopia's Path towards Universal Health Coverage through Strengthening Primary Health Care (2015–2035)” (23).

The Ethiopian government has expressed its commitment to help the UHC get grounded in the health system starting from the time when UHC was declared as one of the pillars of the SDGs in 2015. In the envisioning document, UHC is stated as a direction that Ethiopia follows in its health sector transformation to achieve the best health outcomes that would be expected of a lower middle-income country by 2025 and the median health outcomes of an upper middle-income country by 2035. With that intention, UHC is underscored in other Ethiopian national health policy documents too, including the HSTP I and II (first and second phase of the ‘envisioning’) and essential health service package (ESHP) II (24).

**Figure 2:**
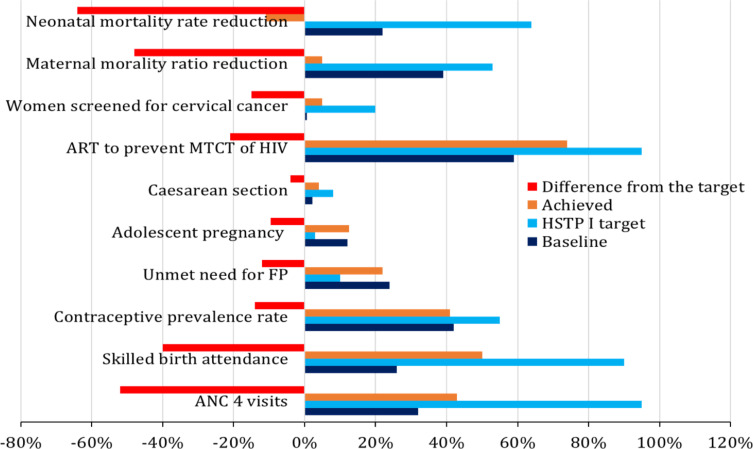
The difference between the HSTP I targets for 2020 and achievements. The baselines for maternal mortality ratio (MMR) and neonatal mortality rate are the difference in percent between 2011 and 2016 EDHS report

The MOH has developed an ambitious plan (HSTP II) for 2024 and 2029 (25). For 11 out of 17 selected SRHR indicators, the plan is to increase each indicator by more than 50% in the coming 10 years. However, as shown in Figure 2, the HSTP I achievements in five years were too far from the target. In declining order, the progress in providing antiretroviral treatment (ART) for pregnant women to prevent mother-to-child transmission (MTCT) of HIV, Contraceptive Prevalence Rate (CPR), and skilled birth attendance were much progressive than other indicators, but neither was close to the set targets.

The national Harmful Traditional Practices Strategy (HTP) launched in 2013 by the Ministry of Women, Children and Youth Affairs (MoWCYA) was the primary policy document of the Government of Ethiopia addressing FGM, child marriage, abduction, and many more (20). This initiative has been observed galvanizing many development partners to join the government's initiative of ending such traditional practices the sooner possible. National Costed Roadmap to end child marriage and FGM in Ethiopia in the Period of 2020–2024 is another policy document launched by the MoWCYA (26).

The government of Ethiopia has also targeted abandoning FGM by 2025. As noted hereunder, however, the pace of the abandonment of this practice is not on track to meet the government's goal. Though there are multiple interconnected factors, the lack of prioritizing such women's and girls' rights and health issues in the health policy might be a contributing factor for the slow progress of abandoning such centuries-old traditions. The sharp decline in abortion-related maternal mortality (from the top for decades to fifth in less than a decade of the abortion law in 2005) (27) is solid evidence how much policy and strategic plan can change the curve.

Ethiopia aimed at achieving UHC by increasing and strengthening the PHC coverage, which is strategically a global approach. A Health Benefit Plan (HBP) comes to the health system in the form of health insurance to maximize the financial protection and ensure that essential health services reach the population equitably and timely. The HBP primarily targets the PHC units (including primary hospitals, health centers, health post, and clinics). There are two health insurance systems under the process of implementation in Ethiopia (Community-based Health Insurance (CBHI) and Social Health Insurance (SHI)) as a primary financial scheme for mobilizing health resources and increasing financial protection and eventually achieving UHC.

## The Status of Comprehensive SRHR Benefit Package in Ethiopia

For several decades, vertical programs, mainly focusing on curative and some preventive SRH services, have been the focus of the health sector globally, for which Ethiopia was not an exception. As a proxy indicator, the share of curative health care services was more than half of the government's recurrent health spending (51%) (28). Specific to SRHR, maternal and child health and Family Planning (FP), have been the top priorities for decades. The WHO has noted that, with the exception of maternal health and FP, SRH services were not explicitly recognized in the health benefits package of many countries, which has led to inequitable access to other critical SRH services (29).

In most Eastern and Southern African countries, CSE, GBV, sexual health wellbeing, and harmful traditional practices such as FGM and child marriage were not prioritized (30). In Ethiopia too, they were not intended to fall into the administrative and service domain of the MOH and Regional Health Bureaus (RHB) as presented in Annex 2 (21–24).

As per their chronological introduction to the health sector domain, relatively advanced services and strong partnerships have been established for 1) providing skilled service to pregnant, delivering and postpartum women; 2) providing modern contraceptive methods; 3) preventing and treating HIV. As a result, the achievements in maternal, perinatal, and child health over the last two decades are remarkable. However, Ethiopia is still far from ending preventable maternal and early neonatal deaths, unmet contraceptive need, FGM, and child marriage. Apart from the lack of focus and strategy, the absence of professionals in the fields of CSE and sexual health is critical.

A meta-analysis has shown that nearly half of Ethiopian women experience lifetime GBV (31). Despite the huge problem across the nation, little is done on the prevention and managing the sexual and GBV victims; only five hospitals in the country have dedicated separate clinic for sexual and GBV victims' clinical service. In other public health facilities, it was observed that survivors of sexual violence are managed like any other patients without giving much time to counseling and psychological rehabilitation.

## Maternal and Child Health Status Indicators

**Figure 3:**
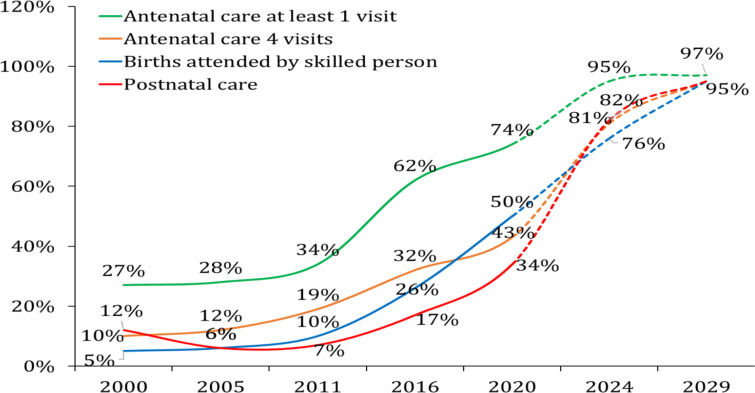
The trend of antenatal care 4 visits, skilled delivery attendance, and postnatal care. Data source: EDHS 2000–2019 and HSTP II projection for 2024 and 2029

According to the EDHS (1995–2019), Antenatal Care (ANC) with at least 4 visits has increased by more than 4-fold. During the same period, the proportion of skilled person attended delivery has increased by 10-fold. The MOH is encouraged by these achievements to increase both indicators to 95% before the SDGs due date. However, as the experience of HSTP I has shown and the multiple factors limiting the acceleration towards the universal coverage of the maternal health services, increasing these two indicators by more than 90% in about 10-year period looks improbable (Figure 3). In practical terms, the HSTP I target for 2020 for ANC 4 visits and skilled delivery were nearly twice higher (90% and 84%) than the recent estimates/achievements (43% and 50%, respectively). By design, having an ambitious plan may urge for better resource allocation and striving to accomplish the near target, but should have been weighed against the reality (Annex 3).

The proportional drop in Maternal Mortality Ratio (MMR), with increasing antenatal care and skilled delivery, is another indicator for the demonstrable collective effect of the SRH services provided in the course of pregnancy and post delivery period. According to WHO estimates, the MMR of Ethiopia declined from 1250 in 1990 to 353 per 100,000 live births in 2015 (32,33). As per the EDHS data, the MMR has decreased by more than half in 20-year period (from 871 to 412) between 2000 and 2016, but there was an overlap of 95% confidence intervals between successive 5-year period MMR reports. The HSTP II MMR target for the SDGs is 140, which is half of the global target <70 per 100,000 live births (34). Still the little change in MMR between 2015 and 2020 (a reduction from 412 to 401, while the target in the HSTP I was 199) may make the 65% reduction by 2029 a bit unrealistic. It is also recalled that the MMR goal of Ethiopia for the MDGs was 267 per 100,000 live births, while the achievement was by more than half less.

As small-scale studies and clinicians' day to day observation have shown, a dramatic reduction in obstructed labor is a solid fact over the last decade. As a result, these days, the occurrence of new obstetric fistula is extremely low. Until proved otherwise with a national survey, the MOH plan of “Ending Fistula and Transforming Lives by 2020” by reducing the incidence of obstetric fistula among women with obstructed labor to <1% is probably already achieved. According to EDHS, the prevalence of obstetric fistula has reduced from 1% in 2005 to 0.4% in 2016. The recent estimate during the obstetric fistula strategic plan (2021–2025) development is 0.03% of the total estimated deliveries. Due to the lack of cases coming to the nine fistula treatment centers, their four years' performance was 4-fold less than the plan.

**Figure 4:**
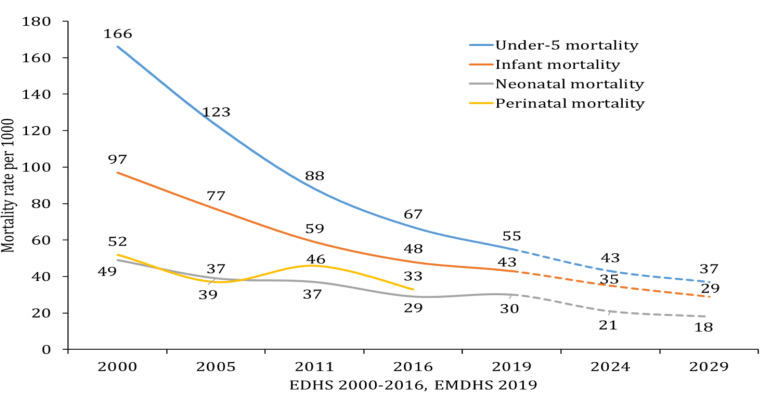
The trend of under-5, infant & neonatal mortality per 1000 live births, and perinatal mortality per 1000 total births after 28 weeks' gestation over 20 years in Ethiopia. Data source: EDHS and HSTP II

Another remarkable progress made is improvement of child health. Analysis of a series of EDHS data has shown a reduction of nearly 67% in under-5 mortality, 56% in infant mortality, 39% in neonatal mortality, and 37% in perinatal mortality rates. Many agreed that this achievement is primarily a result of the intensive work of the public health sector and development partners on immunization. However, there was no significant decline in early neonatal mortality over the decades, which is contributing to the relatively lower reduction in neonatal and perinatal mortality rate (Figure 2). Nonetheless, the progressive decline is probably what has persuaded the MOH to aspire a reduction of neonatal mortality rate to 18 per 1000 live births (Figure 4), which is close to the SDG global target (12 per 1000 live births) (2).

## Contraceptive Methods Utilization

FP service is another component of the SRHR services, which has shown remarkable progress in Ethiopia in the last decade. Modern CPR has increased by more than 5-fold in the last 20 years. The other side of the coin is that the unmet contraceptive need has been reduced by 15%. The birth spacing, avoiding unplanned pregnancy, and thereby unsafe abortion with contraception is presumed as major contributors to the maternal and child health improvement noted above.

**Figure 5:**
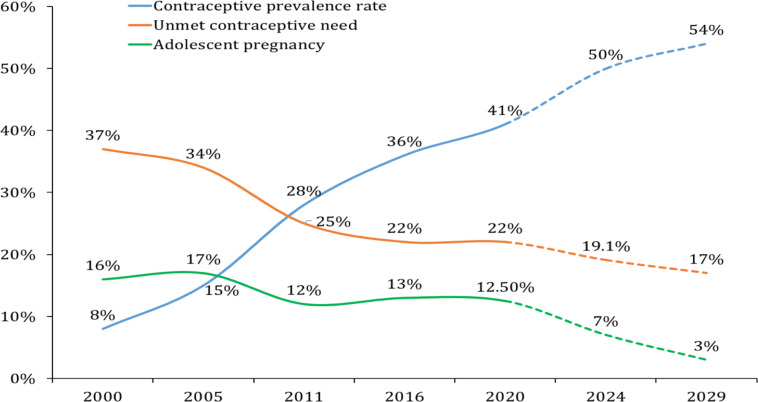
The trend of contraceptive use and unmet need by married women, and the proportion of teenage/adolescent pregnancy in the last 20 years and the projection in the coming 10 years. Data source: EDHS 2000, 2005, 2011, 2016, HSTP I and II.

As a proxy indicator for several determinant factors, including contraceptive utilization, the proportion of teenage pregnancy, however, remains in the high range (Figure 5). This is partly the reflection of the high child marriage (noted below) in many parts of the regional states, the low utilization of contraceptives, and early sexual debut regardless of the details. HSTP II target is to reduce the proportion of teenage pregnancy to 3%, which was also the case in HSTP I and Reproductive Health Strategy (2016–2020) (35), but has not shown much improvement. Child marriage is still a staggering figure, particularly in Northern and North Western part of the country.

## Status of PMTCT of HIV

Overall, although new HIV infection is still a huge problem (14,843 in 2019, including 3,229 children aged less than 15 years), the HIV prevention and treatment program is assessed as relatively successful (estimated national prevalence among people aged 15–49 years is 0.9%) (24). In the interest of this review, the PMTCT of HIV is far from the state of elimination (<5% incidence of MTCT of HIV among breastfed). The estimated prevalence of MTCT of HIV was in the range of 5.6–13.4% (21,34), According to Ethiopian Public Health Institute (EPHI) 2019 estimate, there were 19,110 HIV positive mothers, of which only 74% had received antiretroviral drugs (ARV). In the same year, according to the MOH annual report, the test and postnatal dual ARV prophylaxis uptake of HIV exposed infants were 64% and 53%, respectively. The low coverage of the ANC, institutional delivery, and postnatal care is attributed to such low performance. Thus, MOH launched a triple elimination of MTCT of HIV, syphilis, and hepatitis B virus strategy for the period of 2021–2025 with due emphasis to testing and treating by improving the continuum of maternal care.

## Progress Towards Ending FGM and Child Marriage

Among more than 140 types of HTP in Ethiopia, FGM, early/child marriage, marriage by abduction, and wife beating are the commonest, which belong to the domain of SRHR issues. Other SRHR related issues commonly violated/practiced are exchange marriage, arranged marriage, widow inheritance, isolating girls and women during menstruation and delivery in a bush, and applying lip disk to increase marriageability (20,26,31).

**Figure 6:**
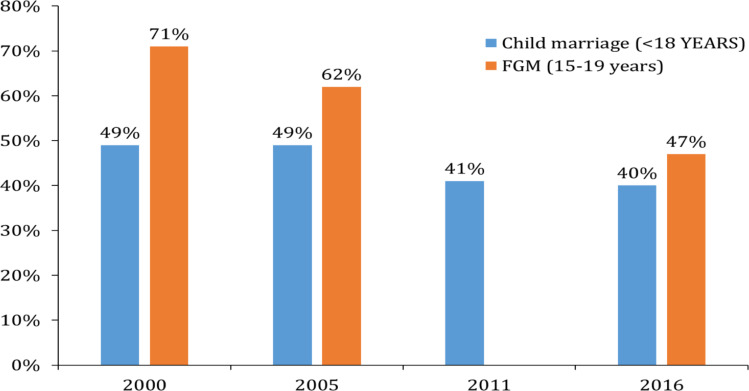
The trend of FGM and child marriage in women aged 15–19 years and 20 – 24 years in Ethiopia, respectively. Data source: EDHS 2000, 2005, 2011, and 2016. Note that FGM was not reported in EDHS 2011

Although the government of Ethiopia refreshed its commitment at the London Global Girls' Summit held in July 2014 to end FGM and child marriage by 2025, as the series of EDHS reports showed, the progress is far from the target (Figure 6), particularly in Ethiopian Somali and Afar regional states, where 99% and 91% of women aged 15–49 years had undergone FGM, respectively. Nevertheless, the decline in the national FGM prevalence rate among young females by 24% in about 20 years (EDHS 1995–2016) and 16% prevalence in children aged 0–14 years are encouraging achievements. In essence, the number of girls and women being cut is decreasing, but not rapidly to reach the elimination level. With the constitutional definition of children (<18 years), the progress in reducing child marriage is too little; there is an overlap between the 2000 and 2016 proportion of child marriage confidence intervals, which indicates the lack of a significant decline. In general, as learned from the previous experience, ending child marriage and FGM may take us a long way unless progressive and intensive community-centered programs are implemented, and much is worked on educating girls.

## SRHR Services Included in the Current Ethiopian Financing and Financial Protection Arrangements

The WHO report has shown that accelerating UHC cannot be achieved without increasing financial protection and without inclusion of essential health services, which is also applicable to universal SRHR services (37). In many low-income countries, including Ethiopia, however, financing the SRHR services and others is still a major challenge for achieving universal SRHR services within the scheme of UHC, partly attributed to financial constraint.

Beyond income, the challenge is probably due to lack of or immature health financing system to generate sufficient and sustainable health finance, leading to inaccessible, inefficient, and poor quality of health services (38). The WHO noted that the success of health financing relies on the health systems capacity to generate revenue, share risk, and implement a strategic purchasing of quality health services (39).

Probably, with due consideration of these health financing values, the financial arrangement/schemes in the EHSP II of Ethiopia are planned to be: 1) cost-recovery, 2) cost-sharing, and 3) exempted services (Annex 4). The SRHR and other health services are grouped under these three categories, and the EHSP II provides a lot of offer free of charge for some of the SRHR services, including safe abortion and post abortion care, medications, procedures, and ectopic pregnancy management, progressing pregnancy and neonatal care (routine to major procedures and medications), CSE, and counseling on sexual health and treatment of menstrual problems. However, this review could not find evidence practically demonstrating the inclusion of CSE and sexual health and wellbeing services in the health sector programs. Similarly, in one study, nearly 60% of included low-income countries did not include CSE, infertility, safe abortion, reproductive cancers, and GBV in their SRHR benefit package (30). A global review has shown that CSE has a positive impact and reduces the risk of vulnerability to HIV/STI, unintended pregnancy, GBV, and early marriage (16).

Among the reproductive cancers, it is only cervical cancer prevention, which is given due consideration in the last five years. Even that is not yet scaled up to reach the majority of the eligible girls for vaccination and women for precancerous lesion screening. Regarding making diagnosis and treatment of all reproductive cancers, there is little offer to bring about financial protection, invariably not that much different from the traditional and routinely practiced health services. The same is true for sub-fertility and infertility treatment. The inclusion of such services into the full financial protection schemes may take a long time, and is likely to be determined by the muscle of the domestic financing.

## The Prospect of Including All SRHR Services in the UHC Benefit Package, Financing and Financial Protection Arrangements

It is not debatable that for the inclusion of the remaining SRHR services and making them equitably accessible, still financing and the financial protection arrangements are the major determinants. Financial protection for SRHR services is partly an economic issue, and partly the government's commitment to implement different financing schemes. Currently, the major sources of health financing in Ethiopia are government budgets, external funds from bilateral and multilateral development partners, and out-of-pocket (OOP) expenditures.

Several large health service programs, including maternal and child health, malaria and tuberculosis (TB), and HIV treatment, neglected tropical diseases, are hugely supported by external funds, but may not continue in the years to come. As the country is progressing to the status of low-middle income group, the donors' support is feared to be fading, and the need to elevate the domestic healthcare financing schemes, including CBHI and SHI, is foreseeable. As learned from the experience of middle-income countries, the financing landscape has progressively shifted from external to domestic financing when they continue advancing from low-to middle-income status, initially with plateauing and later declining and ceasing of external health financing (40).

This is also looming in Ethiopia as many of the development partners have placed increasing pressure on self-reliance, particularly after they have noticed the growth rates of the annual Gross Domestic Product (GDP) on average by nearly 10% for about a decade and the projected growth being remained above 8% (41). Such a rate of economic growth opens room for the donors to progressively withdraw, by assuming that it increases the new fiscal space for health by increasing the government's expenditure (essentially by increasing domestic financing), which is instrumental to ensure the sustainability of impactful health programs.

The projection herein demonstrates how the progressive macroeconomic growth (measured by real GDP growth) can increase the new fiscal space for health and persuade the donors' withdrawal. It is projected that the overall government revenues from domestic resources as a percentage of GDP will increase from 14.7% in 2017/18 to 16.3% in 2021/22, which is expected to increase the domestically generated government health expenditure to 2.2 billion USD by 2025. According to experts' estimation, this amount of health expenditure can replace the external funding for health and enable to reduce the OOP expenditure by 15% (42). As a yellow flag for the withdrawal of donors, the global fund report made the following conclusion: “The pervasive perception within higher levels of government that health programs are well-financed with donor support has resulted in hesitance to allocate additional domestically generated resources to the sector” (41).

A significant decline in external funding for key programs (such as HIV, malaria, and FP) in recent years is a very good example. Referring to the World Bank's economic classification of countries, the multilateral fund sources (such as GAVI) clearly stipulated in their bylaws that they will withdraw their support as soon as a low-income country graduates with a Gross National Income (GNI) per capita between 1,026 and 3,955 USD (1500 USD specific to GAVI). According to World Bank data, Ethiopia's GNI per capita in 2019 was 850 USD, and projected to be 1,006 USD by 2025 (43), which is a target for lower middle-income country.

The nominal total health expenditure (capital and recurrent) of Ethiopia has increased by nearly six-fold between 2004/15 and 2016/17 (from 522 million to 3.1 billion USD), but the proportion of development partners' contribution for the increment in later years was much lower than government and OOP expenditures (26,39). The 2016/17 health financing of donors was 15% less from 2010/11. The overall trend is nominally towards slowly increasing domestic financing.

**Figure 7:**
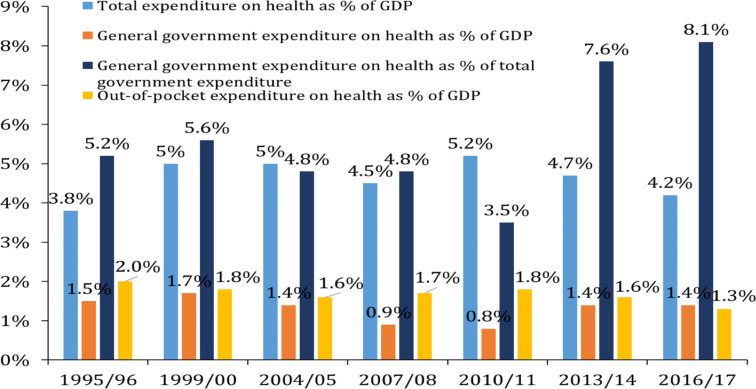
Ethiopian health accounts indicators of healthcare over 20 years. Developed from reference # 24

Of note, although the total health expenditure on health, in particular, has increased by about 6-fold after ten years, the general government expenditure on health as a percentage of total government expenditure was only about 8%, which was marginally lower than the other low-income countries average (8.7%), and nearly less by half of the Abuja Declaration target (15%) set in 2001 by the member States of the African Union (44). In over 20 years (1995/96–2016/17), the total health expenditure, the general government expenditure on health, and OOP expenditure (each as a percentage of GDP) has remained almost being plateau (Figure 7). In 2016/17, the total health expenditure was less than half of the world average (9.2%); less than the average for low-income countries (5%) (44); and even lower than the previous years starting from 1999/2000.

Unless the Government of Ethiopia makes a concerted effort to increase public domestic financing to support the health sector, there may be a risk of resurgence of diseases prevalence/reversal of positive trends in health status indicators. Specific to the interest of this review, the majority of the SRHR services, which are already in the health system, are hugely supported by external donors. The progressively declining external funds and the plateauing of the health expenditure as per the percentage of the GDP are foreseeable risks to advance the SRHR services unless sustainable and growing schemes of domestic health financing are implemented. In 2016/17, the proportions of government, donors, OOP, and others contribution to the total health expenditure were 32%, 35%, 31%, and 2%, respectively, while in 2010/11, the proportions for the first three sources in their order were 16%, 50%, and 34% (45).

Over 20 years, OOP expenditure accounts for nearly or above one-third (31–53%) of the total health expenditure, which was much higher than the global average (21%) and WHO recommendation for low-income countries (20%) (45). Total health expenditure per capita (per person) has reached 33 USD in 2016/17, which is by more than two and half-fold lower than the recommendation for low-income countries (86 USD). According to the WHO, total health expenditure per capita has to be 112 USD to achieve the SDG (46). Overall, until 2016/17, the share of domestic financing has increased to more than 64% (30).

In Ethiopia, the share of spending on SRHR was only 8%, while the share of infectious and parasitic disease prevention and treatment was 53.6% (28). Therefore, so as to progressively include all SRHR services in the UHC benefit package and ensure financial protection, Ethiopia has to increase the domestic financing schemes as elaborated hereunder.

## Establishing a Sustainable Domestic Health Financing System: A Decisive Pathway to Accelerate UHC in Ethiopia

The HSTP II aims further decreasing the OOP expenditure to the WHO recommendation, the government's health expenditure to Abuja declaration, and the per capita health expenditure to 81 USD, by increasing the proportion of eligible households enrolled in CBHI from 49% to 100% coverage to reduce the incidence of catastrophic health spending from 2.1% to 1.5% and ensure the health financing by 2029 (24). Materializing this plan is huge progress to achieve UHC, which is widely accepted as enabling strategy to indiscriminately access quality health services (including SRHR).

To make the health financing sustainable and shows an accelerated increment in both volume and per capita health spending, the domestic financing has to make a landscape shift from the dominance of household OOP health financing to further increase the share of the government expenditure and strengthen the health insurance system. According to the 2016/17 data, the contribution of CBHI as a subset of the OOP health financing was only 1% (29).

Thus, on top of increasing the government's share, increasing the CBHI and SHI contribution to the domestic health financing is imperative, and probably the best strategic action to offset the declining donors' contribution and fill the gap due to increasing public health service demand. CBHI and SHI can further widen the fiscal space to offer a waiver for the indigent segment of the population.

That is why CBHI and SHI schemes are strongly proposed as a primary domestic channel for mobilizing additional financial resources to establish sustainable health financing, and achieve financial protection and thereby UHC. Expanding the CBHI to reach to all *Woredas* and at least 80% of the eligible households, and soon initiating the SHI across the nation should be taken as the top priority for action.

In many low-and middle-income countries, fully implementing the health insurance schemes have served as a basis to provide comprehensive SRHR services, for which few example countries are cited in the introduction section. Essentially, CBHI and SHI are anticipated to serve as a vehicle for progressing towards UHC in Ethiopia and significantly reduce household OOP.

Many recommended implementing innovative financing initiatives (including sin taxes, and airline and airtime levies) to augment the government and health insurances share and widen the basis of the health financing. Beyond securing the required health financing, the availability and quality of essential SRHR services are the major concerns on making the domestic financing strong and sustainable.

In general, the health insurance schemes have a strong potential in creating a health service demanding people. So as to include the remaining components of the SRHR in the UHC benefit package in due course and achieve UHC, establishing a sustainable health financing system is a decisive pathway.

## Key ‘Accelerators’ for Attaining Universal SRHR Services

Here, the point is not only SRHR services universality, but also making good progress towards UHC and SDGs. Among SRHR services, which have got priority and financial protection, the indicators for Ethiopia are much better than before, but not yet to the acceptable levels. For instance, the MMR is still among the highest in the world. The early neonatal mortality rate remains almost plateau for decades. The population growth is by more than 3 million per year (the reflection of the high total fertility rate, high unmet contraceptive need, and high incidence of unintended pregnancy), which is disproportionately higher than the economic growth of the country. The prevalence and incidence of harmful traditional practices targeting sexuality are still staggering.

All these major public health problems are in the domain of PHC. The implication is that a health system that prioritizes and anchors much of the resources to PHC can achieve an improved equity and quality in the majority of the SRHR services, and can also elevate the cost-efficiency. Therefore, much work is needed to accelerate both universality and progress to achieve the set SDGs for SRHR status indicators, primarily by giving emphasis to reach to the majority of the SRHR beneficiary population through the PHC. The current review has identified the following key strategic directions as key ‘accelerators’ for attaining universal SRHR services and making good progress towards UHC and SDG 3 targets.

- **Government commitment and leadership**: Government's commitment to attain universal SRHR services and UHC, and the health policies and programs supporting PHC as primary focus takes the lion's share. Securing adequate finance from the government's treasury and mobilizing additional finance from local and international resources is basic to achieve UHC. Without adequate health financing, achieving universal SRHR services and UHC, and the broader set of SDG 3 targets is highly improbable (46).

**Increasing the financial protection:** Ensuring the sustainability and dominance of domestic financing with low OOP expenditure, and adequately resourcing the PHC is one of the accelerators towards achieving universal and financially protected essential SRHR services.

**Community participation:** Engaging individuals and the community in letting them understand and own the problem is considered as a critical step to institute and scale-up SRHR services, and mobilize adequate resources from the community.

**Empowering women:** Empowering women and girls to control their sexuality and be part of the solution for deep-rooted women's and girl's rights violence, including but not limited to the violence of marriage rights (age-disparate sexual relationships, FGM and lip disk for marriageability, abduction, polygyny), sexual and physical violence against women and girls/boys (beating, harassment, rape), patriarchal norms disregarding women's rights and persuading for gender stereotyping.

**SRHR services integration:** Integration of SRHR services is an important approach to maximize the cost-effectiveness of service provision at a time and minimize missed opportunities. In parallel, prioritizing and promoting SRHR services is one step forward to ensure equity and universal access (29) by implementing a coordinated multisectoral response, particularly to those SRHR services which have got little attention.

**Overarching SRHR framework and measurements:** Aligning the health development partners SRHR activities and support with the public health sector policies, programs, and evaluation so as to reduce duplication of efforts and maximize efficiency is another important undertaking.

**Multi-sectoral response to SRHR services:** As SRHR is not only the responsibility of the health sector, multi-sectoral and multi-stakeholder responses and coordination is another accelerating approach, particularly for assuring women's rights and gender equality.

**Strengthening the humanitarian-development nexus:** Reaching a fragile and vulnerable population is ensuring equity and availing quality SRHR services to those who are hard to reach and most underserved segment of the population.

The government's multidimensional actions, community involvement, focusing mainly on PHC, adequate and sustainable health financing are the major drivers/accelerators of SRHR universalism and UHC.

## Conclusion

The health policy documents of the MOH are supportive and in line with the principles governing the attainment of UHC. This time, a minimum initial SRHR service package is in the pipeline and the experience may serve as a driving force to moving towards offering comprehensive SRHR services in the years to come. Notably, substantive progress is made in family planning service and maternal and child health, for which taking them as a national health priority and providing financial protection for more than a decade have probably contributed a lot. These achievements are the reflection of the government, the health ministry, and development partners' commitment to attain UHC first by improving the health of women and children, as maternal and child health had been taken as some of the MDGs and SDGs indicators.

Nevertheless, the health sector still needs a lot to do to end preventable maternal and early neonatal deaths, unmet contraceptive need, FGM, child marriage, and much more harmful traditional practices targeting sexual health and rights. The lack of including CSE and well-programmed sexual health and wellbeing services, the gray programs for FGM, child marriage, and many more harmful traditional practices in the health sector are the deficits to achieve universal and comprehensive SRHR services in Ethiopia. The top-down administrative health structure to address comprehensive SRHR services is fragmented, and polarized to limited areas of SRHR.

This review underscores that including other SRHR services in due course in the benefits package is possible provided that the domestic financing grows in parallel with the economic and population size growth. Comprehensive and integrated approaches to SRHR with financial protection to essential health services in PHC indicate good progress towards UHC and SDGs. Building resilient health systems through the humanitarian-development nexus for health systems strengthening in fragile setting makes the efforts towards achieving UHC and SDGs complete.

## Recommendation

1. **The MOH should:**
  - Revisit the currently fragmented SRHR management structure and programs within its directorates and other ministries to have a focused, well resourced, and monitored programs
  - Play a spearheading role in the implementation of the strategies, guidelines, and other policy documents of the Ministry. Failure to do so is creating frustration and reservation among the health development partners, and will take us a long way to establish a universal SRHR focused and resilient health system.
  - Prioritize and promote comprehensive SRHR services to create a clear understanding about their significance for the achievement of the UHC and SDGs
  - Include essential package of SRHR interventions through a life course approach, and apply equity in access, quality of care without discrimination, and accountability across implementation.
  - Take the lead in prioritizing CSE and sexual health and wellbeing services; needs to incorporate CSE and sexual health services in its programs and promotes streamlining these services to the health development partners' program portfolio
  - Strengthen the PHC units set up to make the health facilities responsive to the public needs, particularly to those who are registered in the CBHI benefit package
  - Take the lead in creating collaboration and synergy in health system strengthening by approaching development partners and humanitarian agencies through the humanitarian-development nexus platform to respond to the multifaceted humanitarian crisis
  - Take the prevention of FGM, GBV, child marriage and the like as a priority health services, and build a strong collaboration with other ministries (MoWCY, MOE, Ministry of Social Affairs) and development partners to bring about a meaningful impact
  - Develop different financial mobilization schemes and present to the government for approval and implementation
  - Develop a separate UHC roadmap that gives a clear direction on its implementation
2. **The Federal Government and Regional States should:**
  - Show a strong commitment in increasing health budget as a vehicle to accelerate the progress towards the multidimensional SDG
  - Ensure equity and be able to significantly reduce OOP by strengthening the health system of all the blocks and mobilize adequate resources for health
  - Take domestic resource mobilization for health as the top priority to secure and sustain the required budget for financial protection of the essential SRHR services, to fund CBHI premiums and fee-waiver schemes for indigent households
  - Not delay the SHI implementation plan anymore; the government subsidy to the health insurance ‘waiver mechanism’ enables the indigent segment of the population to access quality SRHR services
  - Aim exempting SRHR services (including infertility and reproductive cancers diagnosis and treatment) before 2030 by boosting the health financing, which is also possible in some countries, such as Thailand (47).
  - Ensure equity (at the heart of UHC) and prevent or delay the most expensive health services for disease conditions; resourcing the PHC should be the first and foremost action, primarily targeting health promotion and disease prevention
3. **The MOE:**
  - To make CSE and sexual health services impactful and sustainable, curricula at second- and third-degree levels should be developed and implemented by medical schools, which will be serving as a ground base for initiation of the program at the bachelor level.
  - As per the developed syllabus, SRHR concept should be included in primary and secondary schools' curricula
4. **Ministry of Women, children and youth:**
  - Should strengthen empowering women and girls to make decision on whom and when to get married, when and how many to give birth, to say no for FGM and other GBV by educating, enforcing the existing law, and legislating new statute
5. **Development partners (nongovernmental organizations) should:**
  - Implementing the humanitarian-development nexus approach to reach to the most fragile population in the country and avail the SRHR services should be strengthened
  - As the MOH has expressed and demonstrated its commitment to exempt cost for maternal and neonatal health services, FP, abortion care, much attention should be given to FGM, CSE, and sexual health, and supporting the scale up of the precervical cancer lesion screening
  - Supporting the development of curricula for second and third degree in CSE, and sexual health is probably a quick win
  - Should assist the SRHR stakeholders in conceptualizing and practicing the “rights based approach” as there are misinterpretations of the culturally and legally acceptable concept of the “rights” at the program and facility levels
6. **Comprehensive UHC benefit package:**
  - Pelvic organ prolapse, a disease of the 'poor’ and multiparous, is a huge sexual and reproductive health problem of rural women (55%) in Ethiopia (48), which should be included in the comprehensive UHC benefit package as standalone women's health problem
